# Study on the Response of Ecological Capacity to Land-Use/Cover Change in Wuhan City: A Remote Sensing and GIS Based Approach

**DOI:** 10.1155/2014/794323

**Published:** 2014-08-28

**Authors:** Ying Wang, Xiangmei Li, Jiangfeng Li

**Affiliations:** ^1^School of Public Administration, China University of Geosciences, Wuhan, Hubei 430074, China; ^2^School of Environment, Resource and International Trade, Hubei University of Economics, Wuhan, Hubei 430205, China

## Abstract

This research examined the spatiotemporal patterns of land-use/cover and the dynamics of ecological capacity in response to land-use/cover change in Wuhan city, central China. The data were derived from five years' remote-sensed images, that is, 1990, 1995, 2000, 2005, and 2010. This paper used an integrated approach of remote sensing and GIS techniques, ecological capacity and the bilateral dynamic degree models. The results are as follows. (1) From 1990 to 2010, remarkable changes in land-use/cover have occurred within the studied area, and the most prominent characteristics of the changes were continuous decline of arable land and rapid increase of built-up land. (2) The total ecological capacity dropped from 450.55 × 10^4^ ghm^2^ in 1990 to 447.35 × 10^4^ ghm^2^ in 2010. The eastern, western, and southern parts had higher ecological capacity whereas the northwestern hilly areas and the central district had lower ecological capacity. (3) Due to the conversion from arable land to built-up land, the ecological capacity losses during 1990–1995, 1995–2000, 2000–2005, and 2005–2010 were 155.52 × 10^2^ ghm^2^, 114.12 × 10^2^ ghm^2^, 455.48 × 10^2^ ghm^2^, and 325.26 × 10^2^ ghm^2^, respectively. The study would contribute to better understanding of the effects of land-use dynamics and the evolution of ecological capacity, which can provide scientific basis for land management and environment protection.

## 1. Introduction

Land, as an important carrier of human activity, provides mankind with production sites and means of subsistence. However, with the accelerated process of the urbanization, land-use/cover (abbreviated as LULC hereafter) has undergone remarkable changes, which are closely related to the terrestrial surface material cycles and life-support processes [1]. While contributing to economic growth and certain social benefits, LULC change frequently leads to effects on regional environmental quality, productivity, and adaptability of biodiversity. Breuer et al. [2] pointed out that LULC change affects water balance by interfering with soil-vegetation-atmosphere system. Yang et al. [3] verified that land-use change driven by the Three Gorges Reservoir (TGR) project had great influence on water resources and nonpoint pollutants. Kusimi [4] demonstrated that the Wassa West District of Ghana was faced with climate change, soil erosion, siltation of rivers, and loss in biodiversity due to land-cover change from 1986 to 2002. Li et al. [5] discussed the effect of LULC change, resulting from industrial development on the local ecological environment, and stated that the increased amount of industrial waste water and gas had put a strain on the local environment. Thus, it can be shown that LULC change has significant effects on the ecoenvironment, most of which are detrimental. Given the importance of LULC and its long-term adverse effects on ecological functioning, it is essential to analyze spatiotemporal characteristics of LULC change and quantitatively assess the evolution of ecoenvironment quality associated with LULC change.

Globally, many indices or models have been used to assess the impacts of LUCC on ecological environment. Some reveal the change of ecological pattern through several landscape ecological indices directly, such as number of patches, largest patch index, mean patch area, and contagion index [6, 7]. Others link the state of ecoenvironment and land-use change through indirect models, such as ecosystem services value (ESV) model [8–12], species richness or functional diversity indices [13, 14], and net primary productivity (NPP) model [15]. In addition, Liu et al. [16] built an emergy-based urban system dynamic model to simulate the impact of urban development and economic growth on regional resource consumption and environment conversation. Wu et al. [17] established a hierarchical patch dynamic model to link land-use change with ecosystem processes and explored how LULC change (mainly urbanization) affected ecosystem production and biogeochemical cycling at the local, landscape, and regional scales. However, ecological capacity (abbreviated as EC hereafter) has seldom been used as a tool to explicitly quantify ecological functioning and ecoenvironment changes linked to LULC change. EC is the core concept of the ecological footprint model, which was proposed by Rees [18] and further extended by Wackernagel and Rees [19]. It is a measure of the amount of biologically productive land available to provide the ecosystem services that humanity consumes, and it is viewed as our ecological budget or nature's regenerative capacity [20]. Global Footprint Network initiated its National Footprint Accounts (NFA) program in 2003 to provide scientifically robust and transparent calculations to highlight the relevance of biocapacity limits for decision making [21]. After years of research, theoretical basis and accounting framework of EC have become well improved and simplified. In this paper, by using EC methodology, land of different types in Wuhan can be “translated” into a common unit, namely, bioproductive area, which provides supply ecological goods and services for human activities. Meanwhile, a spatial-temporal assessment analysis is done to measure the changing trend of EC, and transition matrix can provide detailed information about the losses and gains of EC linked to LULC change.

The purpose of this paper was to explore the spatiotemporal dynamics of EC in response to LULC change from 1990 to 2010 in Wuhan city, Hubei province. Recently, studies related to LULC changes in metropolitan areas of China have been focused on east and south coastal regions, such as the Yangtze River Delta, the Pearl River Delta, and the Bohai rim areas. Studies on the spatiotemporal patterns of LULC or the relationship between LULC change and ecoenvironment quality in central areas are relatively scarce. Nowadays, as our national economy development center has begun to shift to central areas from the east, more incentive policies were promulgated. For instance, the Rising Strategy of Central Region [22] was initiated and the Central China Economic Zone (abbreviated as CCEZ hereafter) was set in 2004 to promote the development of central regions. In December 2007, Wuhan city has been approved as the demonstration city of building “the resource-saving and environment-friendly society” (abbreviated as “two types of society” hereafter) by the Chinese government, which would contribute to promoting it to continuously strengthen the comprehensive strength and capability of sustainable development. Since the policies were put into effect, LULC in Wuhan underwent more significant changes due to frequent socioeconomical activities, thus causing more profound impacts on the regional climate, water, soil, plants, and other environment elements [23]. Therefore, the purpose of this study is to examine the spatiotemporal patterns of LULC and the dynamics of EC in response to LULC change from 1990 to 2010. This will lead to a better understanding of land use dynamics and the variation of EC, which might provide scientific basis for land management and environment protection. Moreover, the results from this case study will have practical implications for the other cities in CCEZ and similar cities worldwide.

## 2. Study Area

**Figure 1:**
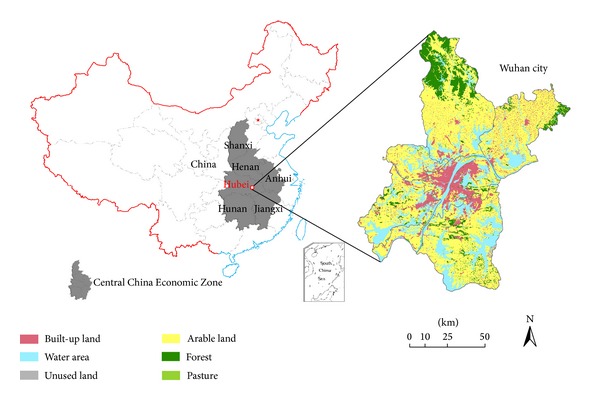
The location of Wuhan city in CCEZ and its landscape.

Wuhan city is located in the central part of China, between 113°41′–115°05′E and 29°58′–31°22′N.  Figure 1 indicates the location of Wuhan city in CCEZ and its landscape. Wuhan covers about 8494.41 km^2^, and the terrain is dominated by plains and supplemented by hills with a surface elevation ranging from 11.3 m to 873.7 m. Landform of Wuhan city belongs to hilly regions in the southeast Hubei province. It is a transitional area between the eastern margin of Jianghan plain and the southern low mountains and hills of Mt. Dabie. The northern subtropical monsoon climate dominates Wuhan year-round, with an annual temperature of approximately 15.8–17.5°C, mean annual rainfall of 1150–1450 mm, and mean annual nonfrost period of 242 days.

Wuhan city has witnessed rapid urbanization and industrialization with a booming economy. In 2010, the GDP per capita in Wuhan amounted to 9,717 US dollars [24], which was much higher than the average GDP per capita (4,946 US dollars) in China. The urbanization level increased from 56.2% in 1990 to 64.7% in 2010. The population density in Wuhan city was up to 985 persons/km^2^ in 2010, which was much higher than the average population density in China (140 persons/km^2^) [24, 25]. However, with the population growth and economic development, LULC in Wuhan has changed dramatically as evidenced by the rapid increase of built-up land at the expense of occupying massive high quality arable lands in the urban fringe areas. Moreover, to gain more construction space, some land exploitation activities such as deforestation and the filling of lakes for the sake of construction space occurred. Compared with other metropolitan cities in China (such as Guangzhou, Shanghai, Suzhou, Shenzhen, and Hangzhou), the ecosystem status of Wuhan was much worse; moreover, Wuhan was in the serious situation of ecological deficit and its ecosystem was quite vulnerable [26].

## 3. Methods

### 3.1. Data Sources and Handling

Time series of satellite data, including five periods' (i.e., 1990, 1995, 2000, 2005, and 2010) datasets of multispectral Landsat TM/ETM+ (path/row, 122/39, 123/38, 123/39) imageries acquired on February 4, 1989, September 2, 1990, April 9 and June 5, 1995, September 13 and September 22, 2000, July 18 and September 11, 2005, and August 9 and September 17, 2010, were selected for this study. All these images were derived from the website of US Geological Survey and the spatial resolution was 30 m. Besides, most of them were from the same season to avoid the confusion in agriculture area and barren land or spatial expansion/reduction of water bodies [27]. An effective interpretation scheme on remote-sensed data was the combination of supervised classification and visual modification and all the procedures were processed using ENVI 5.0 and ArcGIS Desktop10.0 software.

Prior to interpretation, we should decide which land-cover classification system to adopt. There is no internationally accepted land-cover classification system today [28] and the overabundance of land-cover classification systems developed by researchers has resulted in the inability to compare land-cover maps [29]. Herein, to facilitate the comparison with other groups as well as taking into account the needs of our research (i.e., calculating EC), the classification system developed by the Chinese Academy of Sciences [30] was applied and divided the landscape of the study area into six major LULC types: arable land, forest, pasture, water area (including rivers, ponds, reservoirs, and aquaculture waters), built-up land, and unused land.

Primarily, digital images were preprocessed with standard procedures including atmospheric correction, geometric correction, and image enhancement. The images were atmospherically corrected using the ENVI FLASSH atmospheric correction module which was developed jointly by the Air Force Phillips Laboratory, Hanscom AFB and Spectral Sciences, Inc., [31] to remove the atmospheric effects. Then, the GCPs (ground control points) module was applied for making geometric corrections. More than thirty area-distributed ground control points were selected as references on the land-use map created by the second nation-wide land survey (2009). Also, the images were projected to a Gauss-Kruger projection coordinate system with detailed projection parameters as follows: central longitude 114° E, Krasovsky ellipsoid, and false easting 500 km. Furthermore, an image enhancement was performed to increase the visual discrimination between features from the data, and the study area was clipped from the mosaicked images against the municipal boundary layer.

After preprocessing, the first step in the process of supervised classification was to select representative training sites for each LULC type that could be identified in the images. Generally, the Jeffries-Matusita and Transformed Divergence metrics were used to evaluate the class separability [32, 33]. The value of each compared class above 1.80 indicated that the training sites for the two LULC types were well selected while the value below 1.00 suggested that the training sites were hard to distinguish from each other [34]. By merging/eliminating classes with low pair separation, the average pair separations of five periods were 1.87, 1.89, 1.90, 1.94, and 1.93, respectively. The next step was to digitize the entire image around each training site by applying maximum-likelihood classifier, which was widely used in remote sensing because of its robustness [35]. The last step was accuracy assessment and the result maps of land-use status survey in related years were applied. The results showed that Kappa coefficients of the five periods were 0.85, 0.88, 0.85, 0.87, and 0.85, respectively, which met the recommended value suggested by Janssen and van der Wel [36] and the requirement of the research.

### 3.2. Bilateral Dynamic Degree Models of LULC

**Figure 2:**
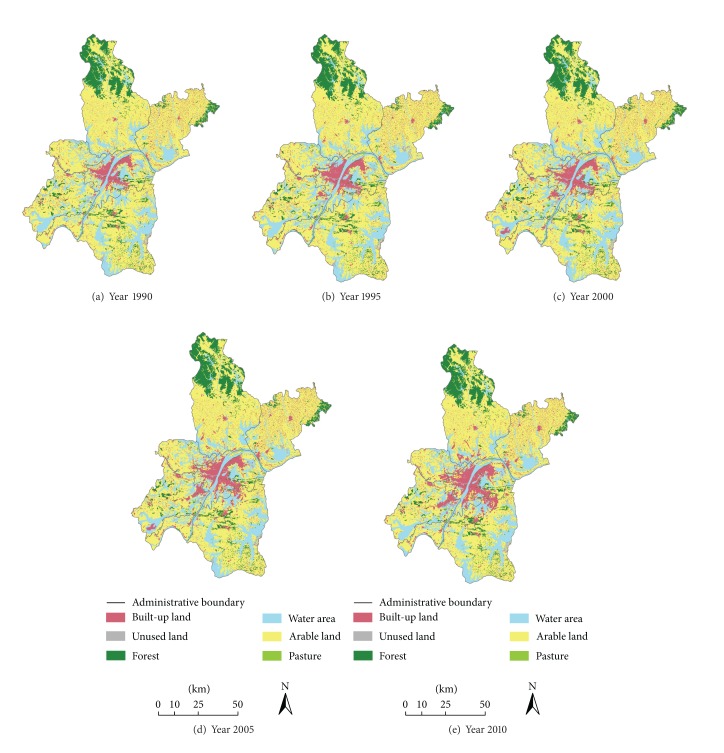
LULC maps of 1990, 1995, 2000, 2005, and 2010 in the Wuhan city.

For exploring LULC change during a certain period, the bilateral dynamic degree model, which was built based on the land-use dynamic model [37, 38], was applied. The model can detect the internal variations and conversions of LULC by estimating the “loss” and “gain” conversions of each LULC type from a given year to the compared year. The five periods' interpreted LULC data (Figure 2) were used, and the formula of bilateral land-use dynamic degree model was as follows:

(1) $$\begin{array}{c} K_{i}=\frac{\sum_{j=1,j\neq i}^{n}\left\{U_{\left(i,j\right)}+U_{\left(j,i\right)}\right\}}{U_{i}}\times \frac{1}{T}\times 100\%, \end{array}$$

where *K*_*i*_ is the bilateral land-use dynamic degree for the *i*th type of land over the monitoring period *T* (five years), *U*_(*i*,*j*)_ is the area converted from *i*th type to *j*th type and represents “loss” conversion, *U*_(*j*,*i*)_ is the area converted from *j*th type of land to *i*th type and represents “gain” conversion, and *U*_*i*_ is the area of *i*th type at the beginning of the monitoring period.

The total difference in the speed and intensity of LULC change for Wuhan city during each monitoring period was calculated as follows:

(2) $$\begin{array}{c} \text{LC}=\frac{\sum_{i=1}^{n}\sum_{j=1,j\neq i}^{n}\left\{U_{\left(i,j\right)}+U_{\left(j,i\right)}\right\}}{\sum_{i=1}^{n}U_{i}}\times \frac{1}{T}\times 100\%, \end{array}$$

where LC is the integrated bilateral dynamic degree of all LULC types.

### 3.3. Ecological Capacity

The ecological footprint methodology uses a common measurement unit to express ecological footprint and EC in terms of a bioproductive area with the global average productivity, introducing the “equivalence factor” and “yield factor.” The equivalence factor represents the world average potential productivity of a given bioproductive area relative to the world average potential productivity of all bioproductive areas. And the yield factors describe the extent to which a biologically productive area in a given country is more (or less) productive than the global average bioproductivity of the same type of land [39]. Hence, by using the equivalence factor and yield factor, land areas with the unit of hectares (hm^2^) can be converted to “bioproductive areas with world average productivity,” and the unit (ghm^2^) is widely used to measure EC. The model of EC can be formulated as

(3) $$\begin{array}{c} \text{BC}=\overset{6}{\underset{j=1}{\sum }}\left(\frac{Q_{j}\cdot Y_{j}\cdot A_{j}}{N}\right)\times \left(100-12\right)\%,\\ \text{TBC}=N\times \text{BC}, \end{array}$$

where *A*_*j*_ is the area of *j*th type of land and *Q*_*j*_ is the equivalence factor for the *j*th type and represents the ratio of the biological productivity of the *j*th type of land to the global average biological productivity for all types of bioproductive land. In the paper, the equivalence factors of arable land, forest, pasture, built-up land, water area, and unused land come from Wackernagel et al. [40], and their equivalence factors are 2.8, 1.1, 0.5, 2.8 0.2, and 1.1, respectively. *Y*_*j*_ is the yield factor for the *j*th type of land and represents the ratio of local (i.e., Wuhan city) biological yield of that type of land to the global average biological yield for the *j*th type of land. According to the value of biological yield of Wuhan city, their yield factors are 2.03, 1.82, 53.53, 1.02, 0.19, and 0.00, respectively. *N* is the number of population in the studied area. BC is per capita EC. TBC is the total EC in the studied area. Taking into account the recommendations of the WCED report “Our Common Future,” 12% of the biological productive area is left for protecting regional biological diversity, particularly for the 30 million species on earth. Herein, 12% of the biological productive area is deducted in the calculation of the EC for biological conservation.

The formula above indicated that EC was determined by the areas of LULC types, equivalence factors, and yield factors. In order to eliminate the impact on spatial distribution pattern of EC brought by patch scale, the LULC maps were divided into 1 km × 1 km grids using ESRI's ArcGIS spatial analyst module. Then, the EC of each grid can be estimated with the field calculator tool of ArcGIS based on the formula.

## 4. Results and Analyses

### 4.1. Dynamics of LULC

**Figure 3:**
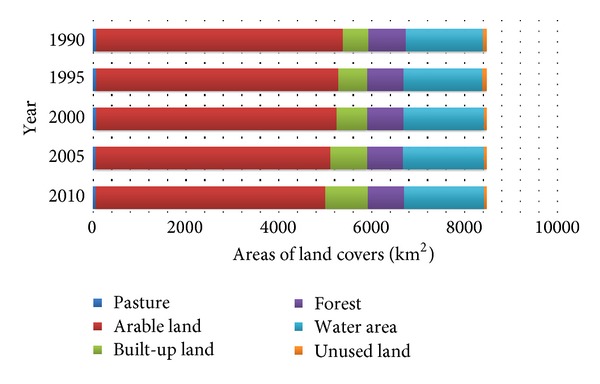
LULC in the Wuhan city from 1990 to 2010.

From Figures 2 and 3, LULC has changed significantly from 1990 to 2010. For example, built-up land underwent impressive expansion from 553 km^2^ to 909 km^2^ within the two decades. Water area also grew moderately while the areas of arable land, pasture, and unused land showed continuous decrease trends. Specifically, arable land decreased from 5322 km^2^ in 1990 to 4950 km^2^ in 2000, reduced by 372 km^2^. The area of forest was relatively stable at around 800 km^2^.

Based on the bilateral dynamic degree models, LULC change rates and the conversions of LULC types during four periods (1990–1995, 1995–2000, 2000–2005, and 2005–2010) were statistically analyzed (Table 1). According to the results of integrated bilateral dynamic degree, the overall change speed and intensity were relatively slow over the former two periods (4.78% and 4.83%, resp.), but the changes accelerated after entering the new century and peaked at 15.89% between 2000 and 2005 and then fell back to 6.31% during the last period. The trend of the change was caused mainly by the dynamic changes of built-up land and unused land. More specifically, bilateral dynamic degrees of built-up land were relatively high throughout the monitoring period from 1990 to 2010 and reached the peak at 43.63% in 2000–2005. Significant increases in built-up land from other LULC types (31.50%) exceeded more than the conversion from the built-up land to other types (12.13%), and the newly emerging built-up land was mainly converted from arable land and water area. The trend was mainly triggered by the increasing demand for nonagricultural land because of urban and manufacturing development [41]. Also, as built-up land was more profitable than traditional agricultural land [42], those profit-oriented local governments and farmers were willing to offer more agricultural land for construction purposes, which accelerated the loss of arable land. Likewise, another remarkable change was a decrease in unused land and the bilateral dynamic degree indices were 16.24%, 27.42%, 34.71%, and 24.30%, respectively. However, more “loss” conversions than “gain” conversions could be seen and unused land was mainly converted to arable land and water area. Furthermore, it was noticeable that the remarkable changes happened during the period 2000–2005 for most of the LULC types whereas the conversions of forest with other LULC types were not significant.

### 4.2. Spatial-Temporal Patterns of EC

From Table 2, we can notice that the total EC of Wuhan city climbed from 450.55 × 10^4^ ghm^2^ to the highest point of 451.47 × 10^4^ ghm^2^ by 2000 and then dipped to 447.35 × 10^4^ ghm^2^ in 2010. Meanwhile, its per capita EC dropped from 0.67 ghm^2^ to 0.53 ghm^2^, which were far lower than the average level of the world (1.80 ghm^2^), China (1.0 ghm^2^), and high income countries (3.1 ghm^2^) in 2010 [43]. In addition, Table 2 showed temporal patterns of EC associated with different LULC types. The EC of arable land accounts for 57.2% of the total EC on average, followed by those of water area (35.8%), built-up land (3.9%), and forest (3.1%). Arable land and water area were the dominant EC types, accounting for 93.0% of the total EC on average. By contrast, scarcely any total EC was provided by pasture and unused land. Furthermore, the EC of arable land experienced continuous decline from 302.49 × 10^4^ ghm^2^ to 281.35 × 10^4^ ghm^2^ (a decrease of 7%) whereas that of built-up land increased sharply from 15.71 × 10^4^ ghm^2^ to 25.83 × 10^4^ ghm^2^ (an increase of 64%) across the years.

**Figure 4:**
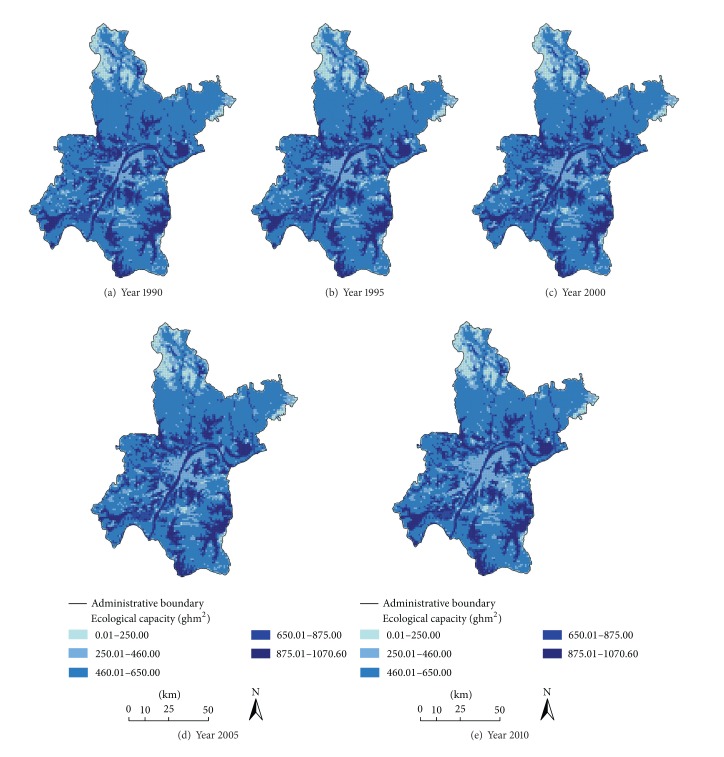
Spatial distribution of total ecological capacity of Wuhan city during 1990–2010.

Figure 4 showed the spatial patterns of EC in Wuhan city from 1990 to 2010. It was clear that the eastern, western, and southern parts of the studied area especially the lands covered by lakes and rivers had higher EC across the studied period. On the contrary, the regions with lower EC were located in the northwestern hilly areas and the central district of Wuhan, which has been an attractor for large population and economical activities. However, rapid development of the economy and population would trigger environmental pollution and ecological deterioration and finally pose threat to regional sustainability [44]. In terms of the variations of spatial patterns, the regions with higher EC surrounding the central district showed a trend of outward diffusion, which indicated the decline of EC in the urban fringe areas. Due to geographic advantages, the fringe areas were hotspots of LULC conversions and most of them were from agricultural types to built-up areas. As different LULC types differed in EC coefficients according to the formula, LULC conversions would trigger EC variations. Hence, the expansion of built-up land which was at the expense of arable land would result in the decline of EC since arable land had higher EC coefficients. Although the total area of Wuhan city would not change, the total EC would vary due to conversions among LULC types. Therefore, results showed that LULC change would exert remarkable influences on ecoenvironment.

### 4.3. Analysis of EC Conversions

For better characterizing the change of EC associated with LULC, a transfer matrix was built. As EC was calculated at grid scale, for each grid, EC was determined by the product of equivalence factor and yield factor. In this paper, the product of equivalence factor and yield factor for each LULC type was defined as “EC coefficient” and a matrix showing the difference of EC coefficients among different LULC types was built (Table 3). The matrix is a table displaying EC coefficients of LULC types in rows and columns. Entries on the diagonal of the matrix indicate that EC would remain unchanged because no transitions of LULC occur, whereas the remaining cells indicate the difference of EC coefficient of a given LULC type that change to a different type. This means that off-diagonal entries display the net change and swap of EC from the conversion of a unit-area land.

To quantify the changes in EC of the studied area, the transfer matrix of EC was created by synthesizing the difference matrix of EC coefficients and the land use transfer matrix [45] of Wuhan (Table 4). Each entry of the matrix is calculated by multiplying the transferred area and the difference of EC coefficient from one LULC type to a different type. As shown in Table 4, along with the political and social-economic factors, conversions of EC and its causes were described as follows.

The EC of arable land increased by 148.03 × 10^2^ ghm^2^, 96.79 × 10^2^ ghm^2^ during the former two periods, but decreased by 12.04 × 10^2^ ghm^2^, 126.74 × 10^2^ ghm^2^ during the latter periods. The gains across the years were mainly attributed to the land conversions from arable land to water area whereas conversions to built-up land were responsible for the losses of EC. Moreover, as the expansion of built-up land accelerated during 2000 to 2010, more losses than gains could be seen. In addition, period 2000–2005 had witnessed a significant increase in conversions from arable land to pasture, forest, and water bodies. The trend was caused mainly by the “Grain for Green” Project (GGP) in 1999, whose objective was to restore China's degraded environment by planting trees or sowing grass on former cropland and enlarge water coverage [46].

Conversions of EC from other types to built-up land far surpassed the reverse process. However, during four periods, conversions to arable land and water area which brought increase of EC were also noticeable, especially after entering the new century. Since China enacted the new Land Administration Law at the end of the 1990s, a series of laws and regulations related to agricultural land protection were made and came into effect, such as the Basic Farmland Protection Regulation (1998), Equilibrium of Requisition-Compensation of Cultivated Land (1999), and Maintain Red Line (the bottom-line area) of the farmland (2008) [47]. The regulations required that any loss of cultivated land be compensated by the generation of a similar area of cultivated land from other land-use types, and the total farmland area should be no less than 1.8 billion mu (120 million hm^2^) in the period to 2020 [48]. Thus, to maintain the dynamic balance of cultivated land, constructed areas such as hollow villages or abandoned industrial and mining facilities were reclaimed to arable land.

There were some other important conversions of EC in response to LULC changes, for example, the conversions between arable land and forest caused by restructuring of agriculture. Specifically, EC of forest increased by 34.90, 6.92, 168.68, and 31.44 × 10^2^ ghm^2^ during four periods after transferring to arable land. Moreover, as unused land converted mainly to water area, accounting for more than 50% of the transferred area, the gains of EC were 31.19, 231.76, 100.26, and 109.42 × 10^2^ ghm^2^, respectively.

## 5. Discussions

### 5.1. The Value of “The Difference Matrix of EC Coefficients”

In order to make further explorations about the effects of land-use changes on ecological environment, this paper puts forward a transfer matrix based on the differences between the product of equivalence factor and yield factor. To simplify the description, the product of equivalence factor and yield factor was defined as “EC coefficient.” The matrix is effective and intuitive and could quantitatively characterize changes in EC caused by conversions of LULC types. With the matrix, we could access the effects of land management policies and LUCC related behaviors and its consequences.

For example, as shown in Table 3, a total area of 1 hm^2^ arable land converted to water area might lead to the rise of EC by 5.02 ghm^2^, but when converted to unused land, pasture, forest, and built-up land, the losses of EC would be 5.68, 5.59, 3.68, and 2.84 ghm^2^, respectively. In contemporary China, urbanization and economic growth have driven more rural labor force to seek employment opportunities in urban areas, and the arable lands which they used to depend on are abandoned. As a result, these valuable lands would convert to pasture and then to barren land spontaneously within several years. Thus, the EC losses of arable land wasting were quantitatively revealed from the matrix. Hence, it provided reference for land management authorities on evaluating the effects of LULC related decisions.

### 5.2. Evaluation of the EC Model

As is well known, our living planet has capacity to provide resources and ecosystem services for economic prosperity and societal well-being, with an ecological limit for human activities. In the ecological footprint (EF) methodology, the EC is defined as the carrying capacity of ecosystems to produce useful biological materials and to absorb waste materials generated by humans. Therefore, EC stands for a more holistic appraisal of regional ecosystems than other measures [49]. In this study, the EC model was applied as a tool to explicitly quantify ecological functioning and ecoenvironment changes linked to LULC change from 1990 to 2010. Through the empirical study in Wuhan city, EC was proved to be a powerful tool for measuring the ecological status of the studied area as follows.

The EC model serves as media to link the status of the ecosystem in a given region with its land-use pattern. Through time series analysis, the EC model presents us with the past and current conditions of the ecosystem, especially in response to LULC change. From spatial perspective, the model converts the land-use pattern to the distribution of EC value, which reflects the spatial difference in the ability of providing ecological goods and services for human activities. To conclude, the EC provides more information about the ecosystem of a region than LULC and serves as important basis for land decisions and ecoenvironment protection.

However, compared with the ecosystem services value (ESV) model, the EC model has far less contents than that of the ESV, not including the gas regulation, climate regulation and the recreation, and culture functions. Future work can be drawn to integrate two indicators in order to reflect the ecological status of complex ecosystem.

## 6. Conclusions

In this paper, we studied the spatiotemporal patterns of LULC and the dynamics of EC in response to LULC change in Wuhan city, central China. The data were derived from five periods' remote-sensed images, that is, 1990, 1995, 2000, 2005, and 2010. The bilateral dynamic degree was applied to characterize the speed and intensity LULC changes, EC was used as a metric of ecoenvironment functioning, and the transition matrices of EC provided detailed information about the losses and gains of EC linked to LULC change. The results showed that from 1990 to 2010 remarkable changes in LULC have occurred within the studied area. Meanwhile, the total EC, per capita EC, and the spatial distribution of EC in Wuhan city have changed significantly due to land conversions among LULC types. The results would lead to a better understanding of land-use dynamics and its ecoenvironment effects, which would offer vital information for land management and environment protection. In particular, it is important to point out that more and effective quantification methods may be adopted to analyze the changing trends of LULC and EC.

Future work should lay more emphasis on the inner driving mechanism of LULC or EC changes and link them with political and social-economic factors by using statistical methods.

## Figures and Tables

**Table 1:** The bilateral dynamic degree of LULC changes from 1990 to 2010 in Wuhan city (unit: %).

Periods	1990–1995	1995–2000	2000–2005	2005–2010
Pasture	5.59	6.30	36.50	16.87
Arable land	3.40	3.01	11.06	4.13
Built-up land	12.38	13.98	43.63	20.38
Forest	2.32	2.17	16.51	1.94
Water area	7.19	6.94	17.79	7.13
Unused land	16.24	27.42	34.71	24.30
Integrated bilateral dynamic degree	4.78	4.83	15.89	6.31

**Table 2:** Changes of ecological capacity from 1990 to 2010 in Wuhan city (10^4^ ghm^2^).

LULC types	1990	1995	2000	2005	2010
Pasture	0.07 (0.0%)	0.07 (0.0%)	0.07 (0.0%)	0.07 (0.0%)	0.06 (0.0%)
Arable land	302.49 (59.1%)	296.58 (58.0%)	294.16 (57.3%)	287.12 (56.2%)	281.35 (55.3%)
Built-up land	15.71 (3.1%)	17.58 (3.4%)	18.75 (3.7%)	22.38 (4.4%)	25.83 (5.1%)
Forest	15.92 (3.1%)	15.79 (3.1%)	15.86 (3.1%)	15.72 (3.1%)	15.64 (3.1%)
Water area	177.80 (34.7%)	181.62 (35.5%)	184.19 (35.9%)	185.27 (36.3%)	185.47 (36.5%)
Unused land	0.0 (0.0%)	0.0 (0.0%)	0.0 (0.0%)	0.0 (0.0%)	0.0 (0.0%)
Biodiversity conservation 12%	61.44	61.40	61.56	61.27	61.00
Total	450.55	450.24	451.47	449.29	447.35
Per capita	0.67	0.63	0.60	0.56	0.53

**Table 3:** The difference matrix of EC coefficients among different LULC types.

LULC types	Pasture	Arable land	Built-up land	Forest	Water area	Unused land
Pasture	—	5.589	2.747	1.907	10.611	−0.095
Arable land	−5.589	—	−2.842	−3.682	5.022	−5.684
Built-up land	−2.747	2.842	—	−0.840	7.864	−2.842
Forest	−1.907	3.682	0.840	—	8.704	−2.002
Water area	−10.611	−5.022	−7.864	−8.704	—	−10.706
Unused land	0.095	5.684	2.842	2.002	10.706	—

**Table 4:** The transfer matrix of ecological capacity from 1990 to 2010 in Wuhan city (unit: 10^2^ ghm^2^).

Periods	LULC types	Pasture	Arable land	Built-up land	Forest	Water area	Unused land	Total
1990–1995	Pasture	—	1.31	2.02	0.42	5.69	0.00	9.45
	Arable land	−5.48	—	−155.52	−18.54	365.11	−43.02	148.03
	Built-up land	−0.05	3.38	—	−0.02	0.16	0.00	3.52
	Forest	−1.05	34.90	1.03	—	6.79	−0.57	42.16
	Water area	−8.20	−135.48	−75.25	−5.69	—	−38.10	−254.53
	Unused land	0.00	0.74	0.78	0.00	29.67	—	31.19

1995–2000	Pasture	—	1.00	4.75	1.06	3.80	0.00	10.60
	Arable land	−1.49	—	−114.12	−31.53	246.61	−4.16	96.79
	Built-up land	−1.64	53.39	—	−0.72	14.83	−0.78	66.73
	Forest	−1.71	6.92	2.56	—	9.94	0.00	19.41
	Water area	−0.23	−145.83	−134.99	−0.16	—	−1.60	−282.59
	Unused land	0.00	39.18	3.24	0.01	189.33	—	231.76

2000–2005	Pasture	—	21.80	5.85	5.89	63.01	−0.01	96.54
	Arable land	−24.92	—	−455.48	−172.62	644.40	−28.35	−12.04
	Built-up land	−1.85	186.28	—	−3.84	62.20	−1.66	242.98
	Forest	−5.04	168.68	9.14	—	74.54	−0.66	251.70
	Water area	−33.13	−509.70	−238.17	−54.94	—	−57.03	−859.84
	Unused land	0.02	30.88	6.51	0.86	62.01	—	100.26

2005–2010	Pasture	—	12.75	4.60	7.24	3.76	0.00	28.34
	Arable land	−2.83	—	−325.26	−0.53	201.81	−2.76	−126.74
	Built-up land	0.00	11.64	—	−0.15	118.28	−1.85	127.93
	Forest	0.00	31.44	0.50	—	5.14	−0.21	36.87
	Water area	−22.76	−179.04	−164.54	0.00	—	−9.41	−352.99
	Unused land	0.08	14.07	6.76	2.92	85.68	—	109.42
